# New Enzyme-Inhibitory Triterpenoid from Marine Macro Brown Alga *Padina boergesenii* Allender & Kraft

**DOI:** 10.3390/md15010019

**Published:** 2017-01-18

**Authors:** Liaqat Ali, Abdul Latif Khan, Muhammad Al-Broumi, Rashid Al-Harrasi, Lubna Al-Kharusi, Javid Hussain, Ahmed Al-Harrasi

**Affiliations:** 1 UoN Chair of Oman’s Medicinal Plants and Marine Natural Products, University of Nizwa, Birkat Al-Mouz, Nizwa-616, Oman; latifepm78@yahoo.co.uk (A.L.K.); albroumi@unizwa.edu.om (M.A.-B.); rashid@unizwa.edu.om (R.A.-H.); javidhej@unizwa.edu.om (J.H.); 2 Marine Science and Fisheries Center, Ministry of Agriculture and Fisheries Resources, Muscat-113, Oman; lubna.alkharusi@gmail.com; 3 Department of Biological Sciences and Chemistry, University of Nizwa, Birkat Al-Mauz, Nizwa-616, Oman

**Keywords:** *Padina boergesenii*, phytochemical investigation, structure elucidation, enzyme inhibition

## Abstract

In continuation to our study of the chemical and biological potential of the secondary metabolites isolated from Omani seaweeds, we investigated a marine brown alga, *Padina boergesenii*. The phytochemical investigation resulted in the isolation of a new secondary metabolite, padinolic acid (**1**), along with some other semi-pure fractions and sub-fractions. The planar structure was confirmed through MS and NMR (1D and 2D) spectral data. The NOESY experiments coupled with the biogenetic consideration were helpful in assigning the stereochemistry in the molecule. Compound **1** was subjected to enzyme inhibition studies using urease, lipid peroxidase, and alpha-glucosidase enzymes. Compound **1** showed low to moderate α-glucosidase and urease enzyme inhibition, respectively, and moderate anti-lipid peroxidation activities. The current study indicates the potential of this seaweed and provides the basis for further investigation.

## 1. Introduction

Man has been using the oceans as a useful resource for producing economically important materials [1]. During the last 45 years, several bioactive secondary metabolites have been isolated and characterized from various marine organisms [2,3]. These secondary metabolites possess a broad spectrum of activities including antitumor, antiviral, and larvicidal, etc. [4,5], and some of them are in various phases of clinical trials [6]. Brown algae are reported to possess a broad range of bioactive carotenoids, polysaccharides, and polyphenols with physiological effects on human health [7,8]. Various secondary metabolites belonging to different classes of compounds have been obtained from brown algal genera and their bioactivities are reported in the literature [9,10,11].

The secondary metabolites from organisms living in the marine environment have been found to possess ameliorative potential in the exploration of drugs for various human-associated diseases. Ailments such as diabetes are regarded serious metabolic disorders in the human body. This is normally shown as high blood sugar, insulin resistance, and a relative lack of insulin [12]. The figures of the World Health Organization suggest that the worldwide occurrence of diabetes is approximately 9%, which is around ~387 million people. Approximately 90% of those with diabetes have type 2, and the severity of the disease led to about 1.5 million deaths from 2012 to 2014 [13]. Diabetic conditions can be suppressed by reducing or blocking the absorption of glucose via inhibiting the related enzymes, such as α-glucosidase. Preventing α-glucosidase enzyme activity reduces the increase in the sugar level of the blood in response to carbohydrate-rich food intake [14,15]. The postprandial counteraction strategies show the reduced rates of oligosaccharide hydrolyses in the small intestine. This in turn slows the frequency of glucose uptake in the blood cells [16]. In such cases, the application of α-glucosidase inhibitors could be an ideal strategy. In this regard, prospects have shown that various terrestrial and aquatic plants possess novel chemical constituents, which could help in inhibiting α-glucosidase activity in diabetic conditions [17]. Previously, aquatic- and/or marine-derived plants have been reported to provide potent resources for finding α-glucosidase inhibitors, as described by one of our previous communications [18] and those by other researchers [19].

**Figure 1:**
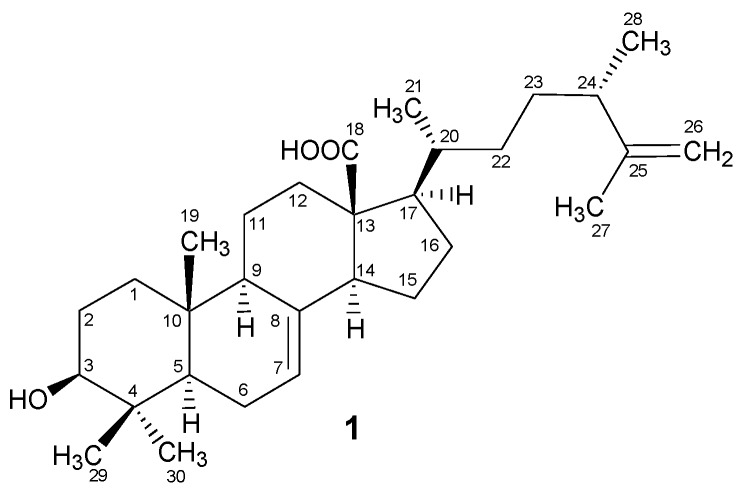
Structure of padinolic acid (**1**).

An important source of antioxidants, brown algae play an important role against lipid peroxidation [8]. The common secondary metabolites of brown algae include long-chain fatty acids, polyphenols, polysaccharides, sterols, triterpenoids, and carotenoids, etc., with toxic and curative physiological effects on human health [7]. A major carotenoid of brown algae is fucoxanthin [20], which is an anticancer agent with chemopreventive effects [21]. The brown algal genus *Padina* is found along the Omani coastal areas, and some of the *Padina* species are considered important sources of iodine and mannitol [22]. The in vivo anticancer activity and the existence of phenolic compounds have already been reported in *P. boergesenii* [23]. Furthermore, in a recent study, the hypoglycemic effect and the Ferric Reducing Ability of Plasma (FRAP) activity of the methanolic extract of *P. boergesenii* was also determined [24]. As phytochemical investigations are carried out rarely on *P. boergesenii*, the present study was conducted to search for secondary metabolites with biological activities. As a result, one new secondary metabolite, padinolic acid (**1**) (Figure 1), was isolated along with some other semi-pure constituents. The chemical structure was elucidated by the combined spectroscopic studies. The new secondary metabolite **1** was also evaluated for potential enzyme-inhibitory activity against lipid peroxidase, α-glucosidase, and urease enzymes.

## 2. Results and Discussion

### 2.1. Structural Characterization

Compound **1** was separated and purified in the form of amorphous powder by using recycling HPLC. The thin layer chromatography (TLC) plates showed a pink color when heated in the presence of cerric sulphate spray. This observation indicated the terpenoid skeleton in **1**. The combined analysis of ^13^C NMR data and FAB-MS proposed the molecular formula C_30_H_48_O_3_ for compound **1**. The FAB-MS data indicated the presence of *pseudo*-molecular ion peaks at *m*/*z* 457 [M + H]^+^ and *m*/*z* 455 [M − H]^−^ for protonated and deprotonated molecular ions, respectively. The molecular formula was further confirmed through high resolution mass spectrometry (HR-ESIMS), which showed the deprotonated molecular ion peak at *m*/*z* 455.3513 (M − 1). The seven indices of hydrogen deficiency were assigned to the tetracyclic system with two olefinic moieties and one carbonyl group in the molecule. These observations were fully supported by ^13^C NMR spectral data.

**Figure 2:**
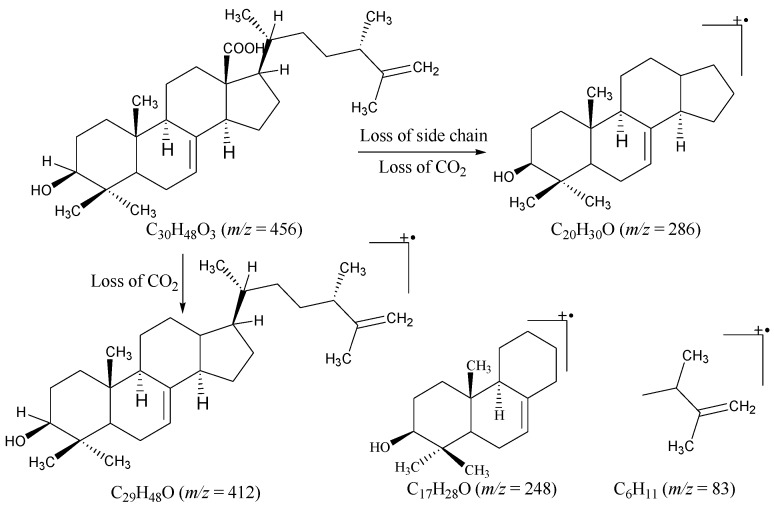
Major MS fragmentation in compound **1**.

The prominent fragments in the EI-MS (Figure 2) at *m*/*z* 133, 203, and 248 indicated the Δ^7^-terpenoid skeleton. The EI-MS also showed fragment ion peaks at *m*/*z* 438, 412, 286, and 83 corresponding to the hydroxyl group, the carboxylic group, and the alkyl chain in compound **1**. The evidence of *retro* Diels-Alder fragmentation was provided by the fragment ion peaks at *m*/*z* 316 and 122, which further supported the Δ^7^-terpenoid skeleton in **1**. The IR spectrum showed the absorptions for a hydroxyl (3460 cm^−1^), a carboxylic acid (1705 cm^−1^), and a trisubstituted double bond (3042, 1625 and 818 cm^−1^). The ^1^H NMR spectrum (Table 1) of **1** indicated the presence of three tertiary methyls and one secondary methyl at δ 0.77 (s, Me-29), 1.03 (s, Me-19), 1.73 (s, Me-27), and 0.88 (d, *J* = 5.5 Hz, Me-21), respectively. Another tertiary (Me-30) and a secondary (Me-28) methyl group appeared as overlap signals at δ 0.97 to 0.99. These methyl groups were further confirmed through ^13^C NMR spectral data, displaying the signals at δ 15.1 (Me-19), 16.1 (Me-29), 16.6 (Me-28), 16.7 (Me-21), 19.5 (Me-27), and 28.6 (Me-30). The olefinic proton (H-7) appeared at δ 5.25 in the form of a multiplet, whereas the H-3 proton (geminal to OH group) appeared as a multiplet at δ 3.16. The terminal methylene protons were observed at δ 4.62 and 4.73 (each d, *J* = 1.6 Hz). These observations were indicative of the terpenoid skeleton in the molecule.

**Figure 3:**
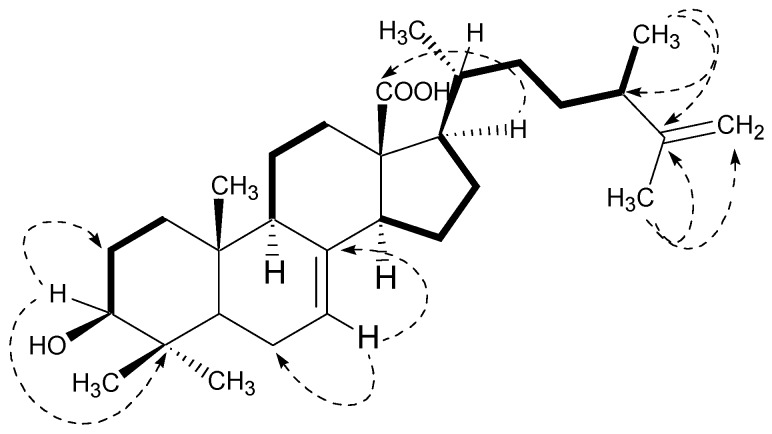
Key COSY (bold) and selected HMBC (arrows) correlations in Compound **1**.

The broadband ^13^C NMR data for compound **1** (Table 1) showed signals for 30 carbons, which were resolved through DEPT experiments into six methyl, 10 methylene, eight methine, and six quaternary carbons. The identity of the side chain was revealed by the signals for one tertiary and two secondary methyl groups along with two methines and three methylenes including a terminal methylene unit. A downfield signal appearing at δ 180.1 was assigned to the quaternary carbon (O=C-18) of the carboxylic moiety, whereas the olefinic carbon C-7 and the hydroxyl-bearing carbon C-3 appeared at δ 126.9 and 79.6, respectively. These observations were also indicative of the Δ^7^-terpenoid skeleton [25,26] which was further supported by HMBC interactions (Figure 3) in compound **1**. The H-3 proton showed HMBC interactions with C-2 (δ 28.0) and C-4 (δ 41.9); H-7 with C-6 (δ 19.4) and C-8 (δ 139.7); H-9 with C-8 (δ 139.7), C-10 (δ 38.3), and C-11 (δ 22.1); the H-14 proton with C-8 (δ 139.7), C-13 (δ 57.5), and C-15 (δ 26.9); and H-17 with C-13 (δ 57.5), C-18 (δ 180.1), and C-20 (δ 39.6), thus providing the evidence for the Δ^7^-terpenoid skeleton as well as the positions of the other groups in the molecule. Hence, the overall structure of compound **1** was found to be similar to the ergostane-type triterpenoids reported from various natural resources [27,28].

After confirmation of the planar structure, the relative stereochemistry of the chiral centers in compound **1** was assigned on the basis of biogenetic consideration coupled with the NOESY experiments. The correlations of H-5, H-9 and H-14 in the NOESY spectrum indicated them to be on the same face of the molecule, whereas the absence of the correlation between H-9 and Me-19 showed the opposite orientation of Me-19 to that of H-9. Hence, coupled with the biogenetic consideration, the *trans* ring junction at C-5/C-10 with the axial orientations of H-5 and Me-19 and the *β*-orientation of Me-19 were confirmed (Figure 1). Furthermore, the correlations in the NOESY spectrum were observed in H-3/Me-30, indicating these substituents to be on the same face of the molecule. The β-equatorial position of the C-3 hydroxyl group was thus confirmed based on the α-axial orientation of H-3 coupled with the *trans* ring-junction at C-5/C-10. Thus, the above spectral studies provided evidence to establish the structure of compound **1** as 3-Hydroxy-4,4-dimethylergosta-7,25-dien-18-oic acid, which has been isolated as a new secondary metabolite. This new compound was named padinolic acid (**1**), in correlation to its producing seaweed *Padina boergesenii*. The chemical structure of compound **1** was similar to the previously reported triterpene sulfates (Antibiotic A-108836) from *Fusarium* species [29]. Compound **1** contains a carboxylic acid group at the C-13 position, and a terminal methylene unit CH_2_-26. The carboxylic moiety is lacking in the known metabolite A-108836 whereas the terminal methylene is CH_2_-28. Furthermore, the hydroxyl at C-3 is in the form of a sulfate, and an additional hydroxyl at C-2 and a methoxy substituent at C-12 are present in the known metabolite [29].

### 2.2. Enzyme Inhibition and Anti-Lipid Peroxidation Studies

Marine algae have adapted themselves to the competitive marine environment [30]. They are considered as the renewable sources of a number of secondary metabolites including cyclic peptides, polyketides, steroids, terpenoids, and alkaloids, etc., possessing a broad spectrum of interesting biological activities [31]. Therefore, compound **1** (10, 50 and 100 μg/mL) isolated from *P. boergesenii* was subjected to enzyme (α-glucosidase and urease) inhibition and lipid peroxidation evaluation.

**Figure 4:**
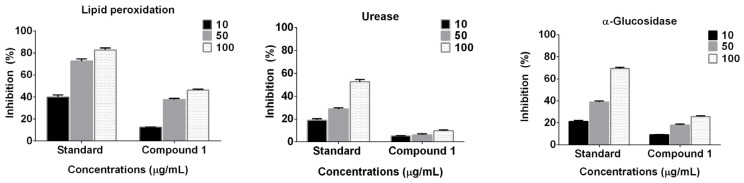
Enzyme (α-glucosidase and urease) inhibition and lipid peroxidation potential of compound **1**; the values in the bar are the means of three replications presented with the standard error.

The results indicated that compound **1** showed a significantly higher (*p* < 0.0001) dose-dependent anti-lipid peroxidation potential (Figure 4). At the maximum concentration (100 μg·mL^−1^), compound **1** showed the highest anti-lipid peroxidation (46.27%; *p* < 0.0001). The two-way ANOVA also suggested that with the increase in the concentration of the compound, the effect is significantly higher (*p* < 0.0005). However, this activity was lower than that of the known standard butyl hydroxy toluene (~82.72%). In the case of α-glucosidase enzyme inhibition, compound **1** showed a dose-dependent activity (10 < 50 < 100 μg·mL^−1^). Furthermore, compound **1** showed a meager suppression of urease enzyme activity. Importantly, both enzyme inhibition patterns were similar; however, the levels of the activities were significantly lower than the standards (acrabose and thiourea; Figure 4).

## 3. Material and Methods

### 3.1. General Experimental Procedures

Optical rotation for compound **1** was measured on a DIP 360 polarimeter (JASCO, Tokyo, Japan). IR spectrum was recorded on a spectrophotometer with ATR-Tensor 37 by Bruker. JMS HX 110 Mass spectrometer (JEOL, Freising, Germany) was used to acquire the mass spectra. ^1^H and ^13^C NMR spectra were recorded on AM 600 NMR spectrometer (Bruker, Fallanden, Switzerland), and the chemical shift values were recorded in ppm (δ) units, whereas the coupling constants (*J*) were reported in Hz. The Final purification of compound **1** was carried out by using recycling HPLC (JAI), and chloroform was used as the mobile phase in a series of two silica columns; JAIGEL-1H and JAIGEL-2H (Serial No. J60-3E13, JAI, Yokohama, Japan). The TLC experiments were carried out on aluminum sheets pre-coated with 60F-254 silica gel (E. Merck, Darmstadt, Germany), which were developed in a mobile phase of 1% MeOH/dichloromethane to 5% MeOH/dichloromethane. The developed TLC plates were then sprayed in ceric sulphate and heated to observe the UV-inactive compounds.

### 3.2. Plant Material

*P. boergesenii* Allender & Kraft (Phaeophyceae) was collected from Gulf of Oman near coastal areas of Sur (22°34′0′′ N; 59°31′44′′ E) during March 2014, and the sample identification was carried out by Dr. Lubna Al-Kharusi, Marine Science and Fisheries Center, Ministry of Agriculture and Fisheries Resources, Sultanate of Oman.

### 3.3. Extraction and Isolation

The fresh seaweed samples were washed with tap water and then freeze-dried. The dried material (750 g) was powdered and extracted with methanol at room temperature to get 110 g of the crude residue. The repeated silica gel column chromatography of the crude extract afforded fourteen fractions at various polarity of *n*-hexane, dichloromethane/*n*-hexane, and methanol/dichloromethane. The sub-fraction #7 (9.5 g), eluted at dichloromethane/methanol (90:10), was further purified through recycling HPLC in a silica column (1H/2H) running in 3.5 mL·min^−1^ flow rate of chloroform (containing 0.5% ethanol as stabilizer). The sample was recycled five times and compound **1** (5.5 mg) was purified with a retention time of 41 min.

#### Padinolic Acid (**1**)

Colorless powder (5.5 mg); ${\left[\alpha \right]}_{D}^{25}$ +5.9 (*c* 0.0007, MeOH); IR (KBr): ν_max_ 3460, 3042, 1705, 1625, and 818. ^1^H NMR (600 MHz, CD_3_OD): See Table 1; ^13^C NMR (150 MHz, CD_3_OD): See Table 1; EI-MS (70 eV): *m*/*z* (rel. int.) 456 [M]^+^ (12), 438 [M^+^ − H_2_O] (3), 412 [M^+^ − CO_2_] (2), 286 (6), 248 (100), 203 (47), 133 (44), 83 (16); (+)FAB-MS: *m*/*z* 457 [M + H]^+^; (−)FAB-MS: *m*/*z* 455 [M − H]^−^; HRESI-MS: *m*/*z* 455.3513 [M − H]^−^ (calcd. for C_30_H_47_O_3_, 455.3520).

### 3.4. Enzyme Inhibition and Anti-Lipid Peroxidation Assay

The α-glucosidase enzyme (E.C.3.2.1.20; from *Saccharomyces cerevisiae*) inhibition assay was performed as described in the method of Oki et al. [32]. The rate of inhibition of the enzyme was measured through ELISA microplate spectrophotometer (xMark, Bio-Rad, Hercules, CA, USA) at 400 nm. The reaction containing *p*-nitrophenyl α-d-glucopyranoside (PNP-G; 0.7 mM) as a substrate, 2.0 units/mL α-glucosidase enzyme and sodium phosphate buffer (50 mM, 100 mM NaCl, pH 7.4) was prepared with or without sample. The reaction initiated by incubation at 37 °C for 30 min. The rate of hydrolysis in PNP-G by α-glucosidase was recorded on ELISA every 30 s.

Besides that, the inhibition potential of the isolated compound for another important enzyme urease was undertaken according to the method of Golbabaei et al. [33]. In brief, a reaction mixture containing urease (Jack bean, Sigma, Hamburg, Germany; 20 mg/mL; 25 μL), urea (100 mM; 55 μL), phosphate buffer (K_2_HPO_4_·3H_2_O 100 mM; EDTA 1.0 mM, LiCl_2_ 10 mM; pH 8.2) and various concentrations of compound (10–100 μg/mL) were incubated at 37 °C for 30 min in 96-well microplate. An indophenol method was used, where the ultimate product (ammonia) was used to monitor urease inhibition activity of the tested compound. The reaction further continued by adding the phenol reagent (1% phenol (*w*/*v*) and 0.005% sodium nitroprusside (*w*/*v*); 45 μL) and alkali reagent (0.1% NaOCl; 0.5% sodium hydroxide; 70 μL) into the wells. The mixture was incubated at 37 °C for 30 min which was followed by recording the absorbance at 630 nm on ELISA microplate reader (xMark, Biorad, Irvine, CA, USA). All the enzyme inhibition assays were repeated three times. The inhibition percentage was calculated through: Inhibitory activity (%) = 100 − (ODtest well/ODcontrol) × 100. Thiourea and acrabose were used as the standard inhibitor for urease and α-glucosidase enzymes, respectively, whereas a blank was used as a negative control. The same concentrations of compound **1** and the standard were used to compare the activity.

In addition to enzyme inhibition, the isolated compound was also analyzed for inhibiting the level of lipid peroxidation. This was performed by a modified method of thiobarbituric acid reactive substances (TBARS) [34]. Using in vitro test conditions, the peroxidation of liposome (phosphatidyl-choline, Sigma, Hamburg, Germany; 50 mg/mL) was initiated by adding iron chloride (0.001 mM; 200 μL), potassium chloride (300 mM; 200 μL) and the test compound (50 μL). The peroxidation was initiated by ascorbic acid (0.001 M; 125 μL), where the reaction mixture was then incubated for 30 min at 37 °C. Trichloroacetic acid (10% and 0.38% TBA) were added. The glass vials containing the reactants were kept at 95 °C in water bath to initiate boiling status for one hour. The resultant change in pink color was recorded on ELISA at 535 nm absorbance. A blank without compound was used as negative control while butyl hydroxy toluene (BHT) was used as positive control. The % inhibition was calculated by using the formula IP% = (1 − A_t_/A_o_) × 100; where A_t_ and A_o_ are the absorbances for compound and control. The experiment was repeated three times.

A two-way ANOVA analysis of the mean values of three replicates was also performed to understand the significance (*p* < 0.05) of concentration vs. activity using GraphPad Prism v6.01 (Software Inc., San Diego, CA, USA).

## 4. Conclusions

The search for new sources of natural products is one of the important steps in the drug discovery process. In the current study, the macroalga *P. boergesenii* was studied comprehensively to investigate the presence of biologically active secondary metabolites. Subsequent to chromatographic extraction and spectroscopic identification, compound **1** was evaluated for potential enzyme inhibition studies. Results indicated that compound **1** possesses low to moderate urease, anti-lipid peroxidation and α-glucosidase inhibition activity, respectively, as compared to the known standards. These findings suggest that seaweed species can be considered as a potential resource for novel bioactive secondary metabolites and future therapeutic agents which cannot be synthesized easily but possess high activity against human ailments.

## Figures and Tables

**Table 1:** ^1^H and ^13^C NMR data (600 and 150 MHz; CD_3_OD) and key HMBC correlations in compound **1**.

C. No.	^13^C (δ)	^1^H (δ)	DEPT	C. No.	^13^C (δ)	^1^H (δ)	DEPT
1	40.1	0.99, m; 1.71, m	CH_2_	16	31.7	1.35, m	CH_2_
2	28.0	1.57, m	CH_2_	17	50.4	1.63, m	CH
3	79.6	3.16, m	CH	18	180.1	-	C
4	41.9	-	C	19	15.1	1.03, s	CH_3_
5	56.9	0.73, m	CH	20	39.6	1.67, m	CH
6	19.4	1.56, m	CH_2_	21	16.7	0.88, d, *J* = 5.5 Hz	CH_3_
7	126.9	5.25, m	CH	22	35.6	1.42, m	CH_2_
8	139.7	-	C	23	33.3	1.52, m	CH_2_
9	54.4	2.21, m	CH	24	40.4	2.33, m	CH
10	38.3	-	C	25	152.0	-	C
11	22.1	1.46, m	CH_2_	26	110.2	4.62/4.73, each d, *J* = 1.6 Hz	CH_2_
12	30.8	1.94, m	CH_2_	27	19.5	1.73, s	CH_3_
13	57.5	-	C	28	16.6	0.99, overlap	CH_3_
14	48.5	3.06, m	CH	29	16.1	0.77, s	CH_3_
15	26.9	1.08, m	CH_2_	30	28.6	0.97, overlap	CH_3_
